# Autumnal Diseases

**Published:** 1859-09

**Authors:** 


### AUTUMNAL DISEASES.

These are diarrhœas, dysenteries, and fevers. Diarrhœa is when the evacuations are thin, frequent and weakening. Dysentery is when there is blood in the discharges, accompanied with a distressing straining without accomplishing anything, called "tormina and tenesmus" by physicians. Fever needs no description.

Diarrhœa, dysentery, fever and ague, bilious fever, congestive fever, typhoid fever, yellow fever, are all one and the same disease, in the opinion of many eminent physicians, differing only in degree, commencing with diarrhœa; this appears earliest in the season, and attacks those who are the weakliest, or are most susceptible of disease.

Those who have a stronger constitution hold out longer, but the causes of disease being still and steadily in operation, their effects are concentrated, and at last manifest themselves in the more aggravated form of dysentery in September.

In October, bilious fevers become the ruling disease.

Persons still more robust, who hold out until November, fall under the terrible congestive chill or typhoid fever, to perish within a few days.

Yellow fever is the result of a more rapid generation of the causes of these ailments, and in a more concentrated or virulent form, but being more speedy in its manifestations, is not, in proportion to the number of persons attacked, as certainly deadly as fevers of the typhoid or congestive type; hence yellow fevers begin in July and August.

Multitudes of lives would be saved every fall, if the people could be induced to give the subject a little examination, and follow it up by the timely observance of a few precautions.

These ailments arise from the decomposition of vegetable matter, requiring however three conditions.

There must be vegetable matter.

There must be moisture.

There must be heat.

When these three conditions meet, a gas is always the result; that gas is called *miasm*, which means an emanation, but it is an emanation of a particular kind—it is that which arises from decaying vegetation alone. The emanations from other things, as a carrion, or a sulphur spring, or privy, are denominated *malaria*—simply "bad air."

Miasm, the destructive emanation from decaying vegetation, as wood, leaves, weeds and the like, has one marked distinctive feature, although a negative one, it has no smell; it is unseen and unfelt; chemistry with all its power cannot detect its presence.

But worse than all this, while the carrion drives us with a power from its neighborhood, miasm not only gives no intimation of its deadly presence, but comes in an atmosphere so cool and so delightfully refreshing, that the temptation to indulge in taking in delicious draughts is as irresistible as the lusciousness of yielding to sleep on the point of being frozen to death.

But here is an apparent contradiction. It is apparent only. Investigation not only confirms the statements, but points out the path of safety, uniform, and infallible.

Miasm is generated by heat of over eighty degrees Fahrenheit, but this so rarifies the atmosphere, that it shoots up into the sky as instantly as an inflated balloon, and as long as the weather continues hot, it is kept among the clouds.

But the cool nights of the fall condense this atmosphere, by which condensation, it descends at sun down to the surface of the earth, where it is breathed until the weather becomes warm enough next day to carry it up again. Hence the popular prejudice against night air.

The Roman authorities do not station officials to caution travellers against stopping in the Campagna during the day time, but in the night, when its swamps are reeking with disease and death.

For the same reason forty years ago the Charleston merchants in summer were not afraid to ride to the city at midday and transact their business, but a night's rest there was almost certain death.

But not to make this article too long for universal quotation which ought to be accorded to it, it suffices to point out its practicalities in all places where autumnal diseases prevail, especially if they are epidemic.

1. Sleep with the outer doors and windows closed, especially if the chamber is on the first floor or story, or even second. This keeps the atmosphere of the room so warm, that the miasm is kept at the ceiling.

2. Take supper at sun-down, and breakfast at day-light, or at least before leaving the house in the morning, even to go outside of the door, or to sit at an open window ; this has the effect to prevent the stomach from absorbing the deadly miasm, as it is pre-occupied by taking something more material and substantial. No doubt the Dutch custom of eating breakfast by day-light, and of the creole, that is the native population of Louisiana, taking their coffee in bed, were founded on observations in this connection without knowing the reason.

3. If a fire is kindled in every dwelling at sun-down, and sun-rise, and the family sit in the same room until bed time, with all outer doors and windows closed, and kept closed during the night, all autumnal diseases, as epidemics, would become impossible of occurrence, because it would be contrary to physical law.

4. A large lump of ice suspended in a sleeper's room, so as to keep the air at the level of his breathing, at seventy-five degrees, would be equally effective in this regard, because miasm cannot be held in solution in an atmosphere of that temperature. It would as it were, be precipitated to the floor of the room, as we know carbonic acid gas is thrown to the floor by a certain degree of cold.

It is greatly to be regretted that these things are not more thoroughly known among physicians, as well as the people, for practical and rational attention to them, would avert an incalculable amount of human suffering.

